# Classification of Bitter Orange Essential Oils According to Fruit Ripening Stage by Untargeted Chemical Profiling and Machine Learning

**DOI:** 10.3390/s18061922

**Published:** 2018-06-13

**Authors:** Saeedeh Taghadomi-Saberi, Sílvia Mas Garcia, Amin Allah Masoumi, Morteza Sadeghi, Santiago Marco

**Affiliations:** 1 Department of Biosystems Engineering, College of Agriculture, Isfahan University of Technology, Isfahan P.O. Box 84156-83111, Iran; s.taghadomi-saberi@ag.iut.ac.ir (S.T.-S.); masoumi@cc.iut.ac.ir (A.A.M.); sadeghimor@cc.iut.ac.ir (M.S.); 2 Signal and Information Processing for Sensing Systems, Institute for Bioengineering of Catalonia, The Barcelona Institute for Science and Technology, Baldiri Reixac 4-8, 08028 Barcelona, Spain; smarco@ibecbarcelona.eu; 3 Department of Electronics and Biomedical Engineering, Universitat de Barcelona, Marti i Franqués 1, 08028 Barcelona, Spain

**Keywords:** bitter orange essential oil, headspace gas chromatography–mass spectrometry, artificial neural network, foodomics, chemometrics, feature selection

## Abstract

The quality and composition of bitter orange essential oils (EOs) strongly depend on the ripening stage of the citrus fruit. The concentration of volatile compounds and consequently its organoleptic perception varies. While this can be detected by trained humans, we propose an objective approach for assessing the bitter orange from the volatile composition of their EO. The method is based on the combined use of headspace gas chromatography–mass spectrometry (HS-GC-MS) and artificial neural networks (ANN) for predictive modeling. Data obtained from the analysis of HS-GC-MS were preprocessed to select relevant peaks in the total ion chromatogram as input features for ANN. Results showed that key volatile compounds have enough predictive power to accurately classify the EO, according to their ripening stage for different applications. A sensitivity analysis detected the key compounds to identify the ripening stage. This study provides a novel strategy for the quality control of bitter orange EO without subjective methods.

## 1. Introduction

Essential oils (EOs) and their volatile fraction have been known since ancient times to have broad applications in prevention and therapy for human health [1]. Undiluted EOs are sold at a high price on the international aromatherapy, perfume, and cosmetic markets [2,3].

EOs from *Citrus* genus are the most popular EOs and are the largest proportion of natural flavors and fragrances [4]. Some orange varieties such as bitter orange (*Citrus aurantium*) are grown primarily for their peel and the associated essential oil industrial production for citrus flavor applications [5]. As an example, EOs from the dried peel of unripe bitter orange fruits flavor drinks and liquors, like Curaçao, Cointreau, and Triple Sec [2]. In the food industry, there is strong interest in their antimicrobial activity linked to the main constituents of their volatile fraction [6,7]. Fruit ripening has the most important impact on the development of flavor and other chemical quality attributes, such as nutritional and biochemical compositions, in citrus fruits. Many researchers have pointed out the importance of ripening on quality attributes of citrus peels and their products such as essential oils [6,8,9]. Owing to the commercial importance of bitter orange EO its characterization and analysis has been extensively developed [10]. For quality assessment, two main types of methods can be used, subjective (sensorial analysis) and objective (analytical methods). The first option is sensorial analysis. This step has several undesired features such as the need of involving a group of trained panelists; this can be problematic for routine analysis [11] as they can suffer adaptation or fatigue [12]. Sensorial analysis can have large sources of variation, low throughput [12], and it can be costly [13]. For this reason, alternative analytical techniques such as separation techniques based on chromatography in tandem with mass spectrometry could be very beneficial; they are non-subjective, highly repeatable, and reproducible. Additionally, the possibility to identify the compounds from mass spectral data is a major factor in favor of this technique [14]. Several studies have already investigated the composition of the volatile compounds of bitter orange EO by this technique. They were conducted to determine chemical families present in the EO [15], authenticity [16], olfactive properties [17,18] or the effects of several factors including geographical location [19,20], season [21,22]**,** and variety, on composition [8,16]. While most research is focused on direct injection of the EO, there are also authors that propose to directly sample the headspace focusing directly on the more volatile fraction [23].

In recent years, there have been an increasing number of studies trying to use the techniques of metabolomics applied to foods. This new approach has been named ‘Foodomics’ [24]. In this strategy, the results of the chromatographic analysis are in many cases further analyzed using chemometrics or machine learning techniques. However, alternative technologies, such as direct injection mass spectrometry and proton transfer reaction time-of-flight mass spectrometry (PTR-TOF-MS) combined with chemometrics methods have recently been used for prediction of sensory profiles [25,26]. For our purpose, the underlying rationale is that EOs can be characterized by a composition fingerprint. While this chromatographic fingerprint [24] will have a certain degree of normal variability, anomalous departures from the normal cluster may indicate voluntary or accidental adulteration. In fact, there is a rich literature in the use of analytical techniques in conjunction with chemometrics for fraud detection or quality determination [19,27,28,29,30,31]. For further information, the reader is referred to the review by Cubero-Leon et al. [31] and the references therein. In the case of citrus essential oils, Parastar et al. used GC-MS fingerprinting in combination with principal component analysis (PCA) and k-Nearest Neighbor classifier (k-NN) to determine from which citrus species (sweet oranges, bitter oranges, lemons, or bergamots) the EO was produced. Additionally, they used counter propagation artificial neural networks (CP-ANN) to determine the chemotypes responsible for this differentiation [32].

A few studies have reported the evolution of the chemical composition of the EO in bitter oranges during the ripening stage [8,9]. They showed that the composition and other properties of the bitter orange EO are subject to important alterations during ripening. The variation levels (%) of chemical classes (monoterpene hydrocarbons, oxygenated monoterpenes, sesquiterpene hydrocarbons, and oxygenated sesquiterpenes) of bitter orange EO do not evolve linearly with the ripening stages [8], and lead to drastic variations in EO quality [33,34]. For this reason, it becomes important to ensure that the EO has been produced with fruits collected at the optimum ripening point. At this point, we should remark that the optimum ripening point may depend on the desired application of the EO; the optimum ripening condition being different for uses such as antioxidant, antifungal, antimicrobial, anti-inflammatory, antiparasitic, green solvent, etc. [2,8,35,36].

The aim of this work is to explore the possibility of controlling the original ripening stages of the citrus fruit from the headspace of the EO using objective methods, avoiding the use of costly human panels. We propose to use headspace gas chromatography–mass spectrometry HS-GC-MS untargeted chemical profiling combined with non-linear classifiers to classify the ripening stage in four classes. Additionally, we will use a feature selection method to determine the most relevant VOCs (Volatile Organic Compounds) for this differentiation.

## 2. Materials and Methods

For the classification of bitter orange EOs, fruits were sampled, and peel oil was then extracted. After sample preparation, the headspace of EOs was analyzed by GC-MS. The total ion chromatogram (TIC) was preprocessed for peak detection and intensity determination. Using selected peaks as the characteristic feature vector, multilayer perceptrons (MLP) were built for sample classification. Additionally, sensitivity analysis (SA) was used for feature selection to detect the compounds more relevant in class differentiation. Using the selected features, final MLP models were constructed for both real and permuted labels. Finally, a permutation test was performed to assess the statistical significance of the performance assessment results. The complete methodology is described in detail in the following subsections.

### 2.1. Experimental

#### 2.1.1. Chemicals and Fruit Sampling

Analytical standard solutions of limonene, myrcene, β-pinene, and GC grade 1-hexanol were purchased from Sigma–Aldrich Corp (St. Louis, MO, USA). Limonene and myrcene standards were used to check the quality of the analytical method (data are not shown). These compounds were chosen since they have been reported as major compounds of bitter orange EO [8,18].

The fruits of the local bitter orange cultivar were taken at four ripening stages (September, October, November, and December) in 2016 from a garden located in Haji Abad, Bandar Abbas (South of Iran). Following literature recommendations, the fruits were sampled depending on the time [5] and color of the bitter oranges [8]. The fruits were collected based on their colors (green, yellow, light orange, and finally orange) with a one-month interval. The pigmented layer (flavedo) of the peel was separated from the soft white layer (albedo) with a sharp knife.

#### 2.1.2. Peel Oil Extractions and Sample Preparation

The bitter orange peel oil was extracted from the flavedo using a steam distillation method [5]. Briefly, flavedo peels were cut into tiny pieces and placed in a round-bottomed flask. Subsequently, the peels were extracted with distilled water in a Clevenger-type apparatus for 8 h. The collected oil was separated from water using a syringe. In this way, 101 EO samples were prepared; 11 from the first stage, 27 from the second, 36 from the third stage, and 27 from the fourth stage. The peel oil samples were stored at −80 °C prior to analysis.

To reduce saturation of detection, samples were diluted with n-hexanol at a ratio of 1:500 (*v*/*v*), and 10 μL of the mixtures were then transferred to the headspace sample vials, which were immediately sealed with a rubber septum.

#### 2.1.3. HS-GC-MS Analyses

HS-GC measurements were conducted in triplicate (three aliquots) at an equilibrium temperature of 110 °C within an equilibrium duration of 10 min. GC-MS analyses were performed on a Focus GC-DSQ II mass spectrometer (Thermo Scientific, Waltham, MA, USA). One milliliter of the samples headspace was injected with a Thermo HS TriPlus 300 headspace autosampler in split mode (split ratio 1:20) at 250 °C. The separation was performed on a 30 m long, 0.25 mm i.d., 0.25 μm film thickness 5% phenyl methyl siloxane HP5 (Agilent Technologies, Palo Alto, CA, USA). A constant carrier gas (helium) flow rate of 1 mL min^−1^ was maintained during the analysis. The following temperature program was used: initially, 70 °C held for 2 min, followed by a 2 °C min^−1^ gradient to 100 °C and held for 3 min, then a 30 °C min^−1^ to 230 °C. The final temperature was held for 3 min. The temperature of the MS transfer line was kept at 250 °C. The electron ionisation (EI) source temperature was 250 °C and the ionization energy was 70 eV. The applied method was based on a modification of a GC-MS method found in the bibliography [37]. Data were acquired in selected ion monitoring (SIM) and full scan mode. In the SIM mode, the fragment ions of hydrocarbons commonly present and odor active in citrus EO (*m*/*z* 136 and 154) and of their isotopes (*m*/*z* 137, 155 and 156) [18,37] were determined after a solvent delay of 0.5 min throughout the run. Mass spectra (*m*/*z* 50–300) were recorded at a rate of two scans per second for identification purposes.

Xcalibur software Version 3.0 was employed for the data acquisition. The data obtained were further processed in MATLAB R2008. The individual compounds were identified by comparing their retention indices relative to analytical standard solutions and by comparing their mass spectra and retention times with data already available in the NIST (National Institute of Standardization and Technology, Gaithersburg, MD, USA) library NIST05 and literature.

### 2.2. Data Analysis

#### 2.2.1. Data Preprocessing

From the raw data, we aim to detect the most relevant peaks and compute their heights. They will be the extracted features for posterior classification with the ANN. For a reliable estimation of the peak intensities, it is necessary to enhance the chromatogram by proper signal processing.

**Figure 1:**
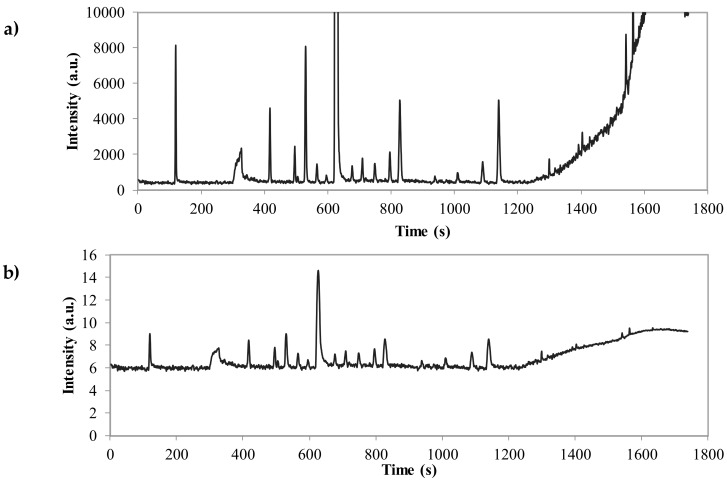

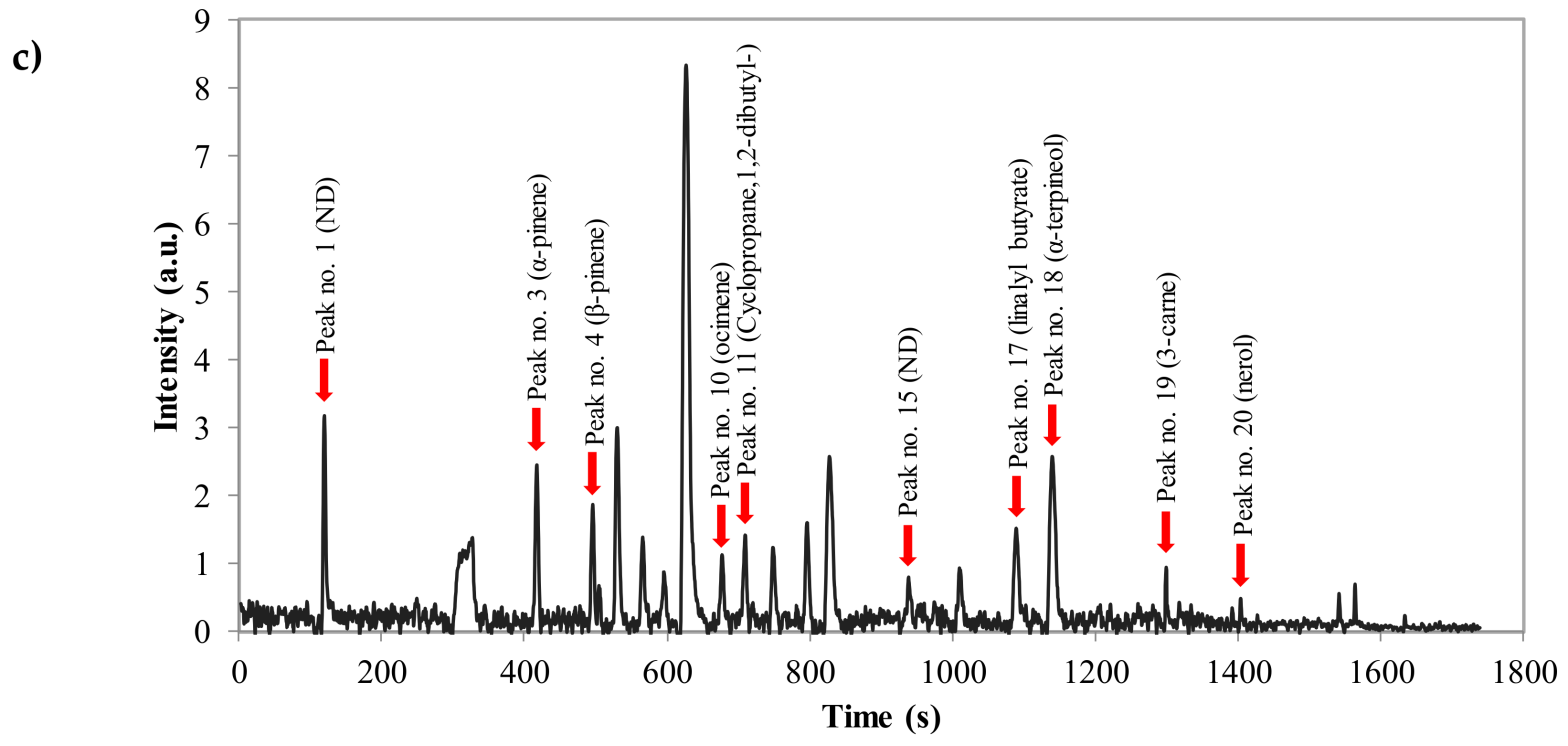
(**a**) Example of a total ion chromatogram (TIC) of bitter orange essential oil; (**b**) logarithmic transformation of TIC; (**c**) TIC corrected by psalsa algorithm, the most effective peaks for classification detected by sensitivity analysis are shown using arrows.

Since the heights of relevant compounds may vary over several orders of magnitude, first a logarithmic transformation of the chromatogram was performed. As depicted in Figure 1a, the limonene peak is considerably higher than other peaks in the chromatograms and the logarithmic transformation has highlighted minor peaks (Figure 1b). The next step was to correct the baseline of the chromatograms. This was carried out using the algorithm named ‘*psalsa*’ (Figure 1c), previously developed by the Reference [38]. This algorithm is a modification of the asymmetric least squares (ALS) baseline removal technique proposed by Eilers and Boelens [39]. Briefly, an adaptive value for the weights of the ALS method depending on the residuals was implemented in order to be more robust in term of parameter variations and to provide more accurate peak intensities [38].

#### 2.2.2. Feature Extraction

In these samples, several peaks related to volatile compounds in the headspace of the EO have intensities that are well beyond the noise level in a consistent manner. A homemade MATLAB routine setting a threshold to peak heights was used to detect relevant peaks and compute their maxima after baseline removal. The selected threshold was determined to be the noise mean plus three times its standard deviation.

#### 2.2.3. Non-Linear Classifier: Artificial Neural Network (ANN)

**Figure 2:**
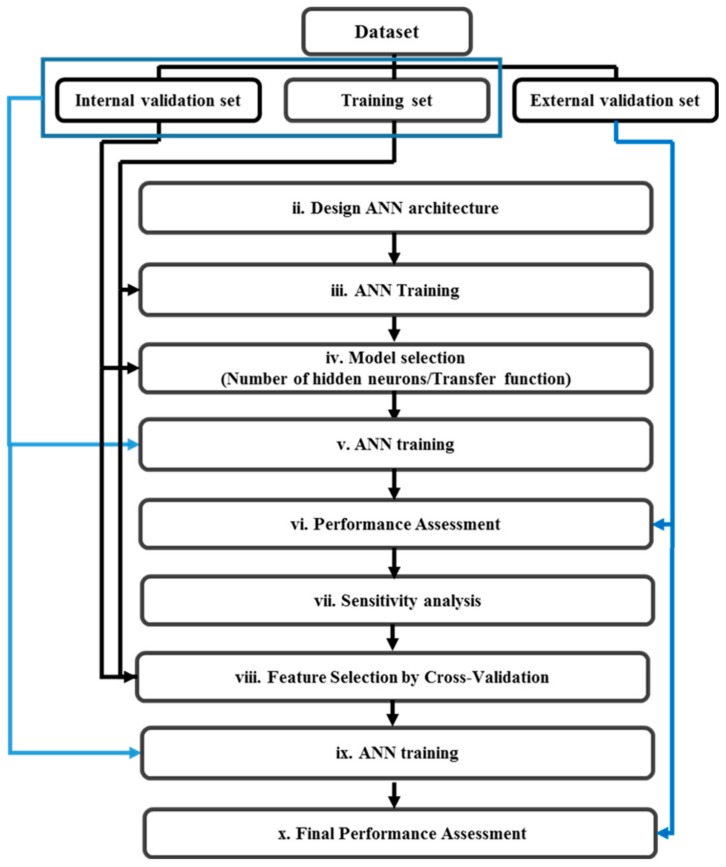
Flowchart for the classifier design and validation including feature selection based on sensitivity analysis.

ANN were used to classify the ripening stages of EOs. Multilayer perceptron (MLP), a common type of ANN for classification purposes [40], was used as the classifier in MATLAB, Neural Network Toolbox. The ability to model complex non-linear input space partitions are among the benefits which are provided by ANNs [41]. For the classification of ripening stages, ANNs were designed, trained, and tested according to the procedure described in Figure 2. The initial data set consisted of 303 samples (101 EOs were measured in triplicate) and 22 features (logarithmic peak heights) as inputs. The MLPs had four outputs; each class of ripening was coded by a four-digit binary number where 1 indicates class membership.

Model selection and assessment were based on double cross-validation (also named cross model validation (CMV) [42]) using a three-way split [43]. A double random subsampling approach was used with 70% for training, 10% for internal validation and model optimization, and 20% for external validation and model assessment (see Figure 2). For precise evaluation of models, some points were considered: (1) all three replicates of each EO were kept at the same subset; (2) the ratio of subsampling for the samples of each class was the same. For example, from 27 samples of the second class, 19 samples (70%) with their replicates were randomly assigned to the training subset in each random subsampling. This strategy was followed in each random subsampling and each subset. Random subsampling was carried out with a total of 1000 partitions.

Different architectures were explored with a different number of hidden neurons and two transfer functions for the hidden layers (hyperbolic tangent sigmoid and logistic sigmoid transfer functions). The linear transfer function for output layer neurons was used (see ii. in Figure 2). Two training algorithms (Levenberg–Marquardt (LM) and resilient backpropagation (RP) [44]) were explored. The goal function to update the parameters of the network was the least squares error (see iii. in Figure 2).

The model selection and model performance assessment were based on the correct classification rate (CCR). CCR is the ratio of the number of correctly classified samples to the total number of samples (CCR = Nright/N) [40]. Model selection was based on the CCR estimated in the internal validation set (see iv. in Figure 2). To obtain parsimonious models, when several architectures provided similar performance, the simplest model was chosen. Once the best ANN architecture with 22 inputs (all peak heights) was chosen, the ANN was retrained fusing the training and internal validation data (see v. in Figure 2). Then, model performance assessment was performed based on the external validation set (see vi. in Figure 2).

#### 2.2.4. Feature Selection by Sensitivity Analysis

The initial feature vector was reduced using feature selection by input sensitivity as goal function. When one or more of the inputs have a relatively small sensitivity in comparison to the others, the input layer of the ANN can be reduced by dropping them, and a smaller-size network can be successfully retrained in most cases [45]. Sensitivity analysis provides extra knowledge on the response of the model to changes in each input. This method was initially developed by Lek et al. [46] and later analyzed in more detail by Gevrey [47]. The response of the best network is inspected by varying each predictor variable in small steps while locking all other input parameters at their mean value [48] (see vii. in Figure 2). Those inputs with a higher sensitivity were retained and those with a lower sensitivity were removed. To determine the optimal size of the input layer for the ANN, an internal cross-validation approach was followed using global classification accuracy as goal function. The search procedure followed a sequential approach by adding peaks according to their best ranking among the four outputs. In the first step, the peaks ranking first for any of the outputs are added, in the second step the peaks ranking second for any output are added. The aforementioned procedure was continued to have all 22 peaks as inputs. The combination of peaks that provides the best classification accuracy is selected.

With the selected features, ANN training (see ix. in Figure 2) and final performance assessment using external validation were carried out again. The new feature vector, consisting of selected features, was fed into the ANNs to train the final models with the same methodology described above (see viii. in Figure 2). To visualize the performance of ANNs, the confusion matrix was created for each model and then averaged over the repetitions (see ix. in Figure 2).

#### 2.2.5. Statistical Significance of the Estimated Accuracy

In order to assess the statistical significance of the obtained CCR values in the external validation set, a permutation test was used [49]. In this test, random labels are assigned to the feature vectors and the whole machine learning procedure is repeated 1000 times. In this way an estimation of the probability distribution of the null hypothesis (H_0_) can be created: H_0_ being the presence of no relationship between feature vectors and labels, and H_1_ being the existence of predictive power from the feature vectors to their real labels. In this way, we can calculate the *p*-value of the obtained CCR taking into account the estimated probability function of the null hypothesis for the CCR [50].

## 3. Results and Discussion

Applying a proper threshold to the data, 22 peaks were consistently detected through all the chromatograms (See Table 1). The heights of the detected peaks of bitter orange EOs were used as the feature vector for the classification.

**Figure 3:**
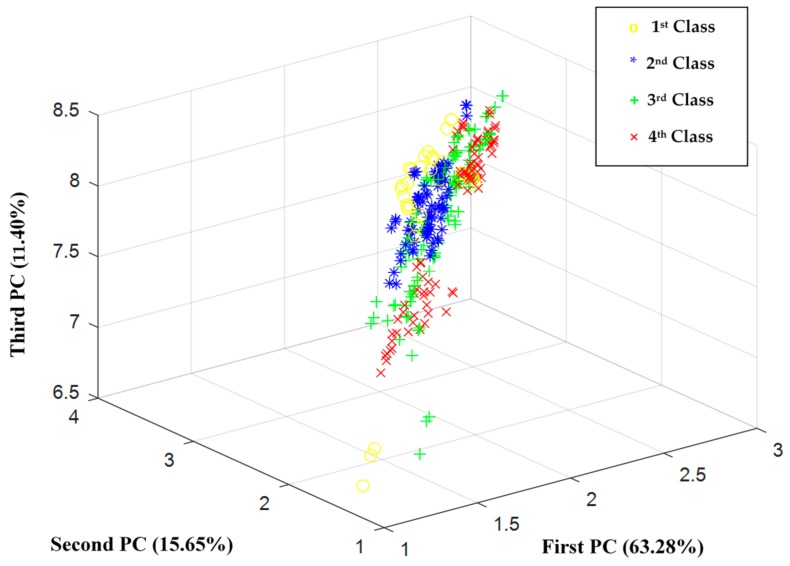
Principal component analysis (PCA) score plot of the volatile components of bitter orange peel oils during fruit ripening.

For exploratory purposes, principal component analysis (PCA) was applied to the feature vector (all 22 peak heights). It was performed for initial exploration of the data distribution and to learn about the inner dimensionality of the dataset since we expected a high correlation among the peak heights. The projection of the data to the first principal components (PCs) did not show a distinct differentiation among the fruit-ripening stages (Figure 3). It indicated that the data separation is not trivial. The total variance of the first three PCs was 90%. Previous research on the changes of the peel EO composition during ripeness confirm our results [8,9]. In other words, there is no specific trend for all compounds in the ripening process; some compounds increase, some decrease, and the others have a changing trend.

Regarding the training algorithm, the RP was evaluated as the best algorithm in comparison with the LM for the classification of ripening stages. Hyperbolic tangent sigmoid and logistic sigmoid activation functions showed no significant difference for the optimizing criterion. The best ANN architecture in internal cross-validation turned out to be 22-4-13-4, that is 4 and 13 hidden neurons in the first and second hidden layers, respectively. The overall accuracy of the model with 22 peak intensities as input was obtained as 70 ± 6%.

**Figure 4:**
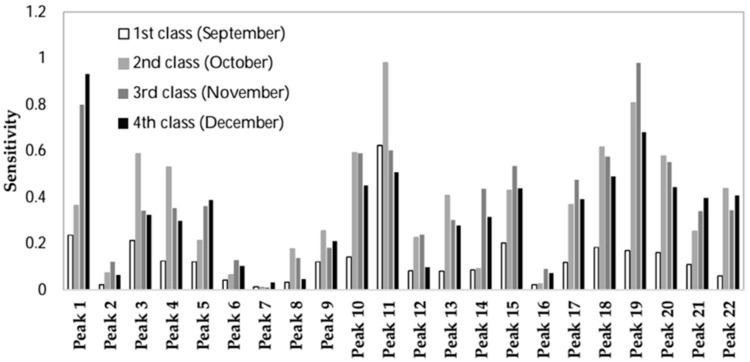
The sensitivity about the mean for the 22 peaks for the different classes.

**Figure 5:**
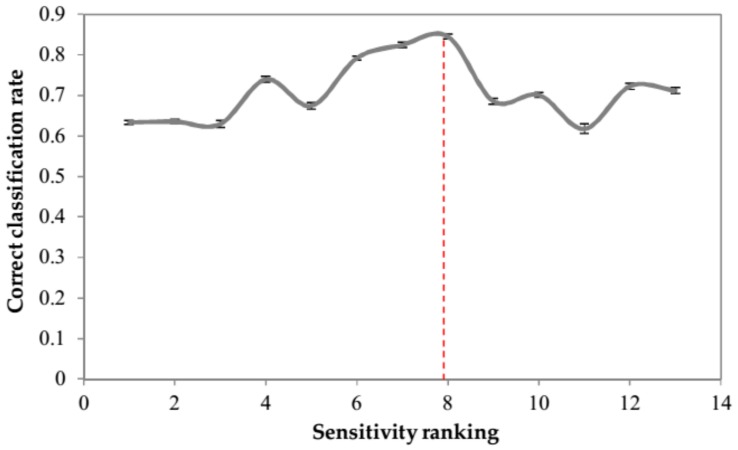
Evolution of the classification rate along the feature search procedure.

The response of the optimum ANN to perturbations made to individual predictor variables (peaks) while locking in all other parameters to their mean value is illustrated in Figure 4. The higher the response (sensitivity) is, the more effective the peak is for predicting each class. Using a sensitivity analysis, the peaks were ranked based on their relevance to predicting each class in Table 2. Peaks number 1, 11, and 19 are the most effective ones for predicting ripening classes. As detailed in the Materials and Methods Section, feature selection was carried out based on the rankings provided by sensitivity analysis combined with prediction accuracy in internal validation based on random subsampling. The classification accuracy in cross-validation is shown in Figure 5. As it can be seen, step viii. including 15 peaks (Table 3) showed the highest CCR. Thus, it was selected as the best set of inputs; including the peaks No. 1 (ND), 3 (α-pinene), 4 (β-pinene), 8 (ocimene), 9 (Cyclopropane,1,2-dibutyl-), 14 (ND), 17 (linalyl butyrate), 18 (α-terpineol), 19 (3-carne), and 20 (nerol). Less sensitivity for other compounds means that their changes do not significantly contribute to output prediction and may therefore be possibly disregarded. Such pruning results in reducing the network complexity and assisting in the network interpretation [51]. Fifteen peaks selected as the most effective ones for modeling the output classes are shown in Figure 1c.

**Figure 6:**
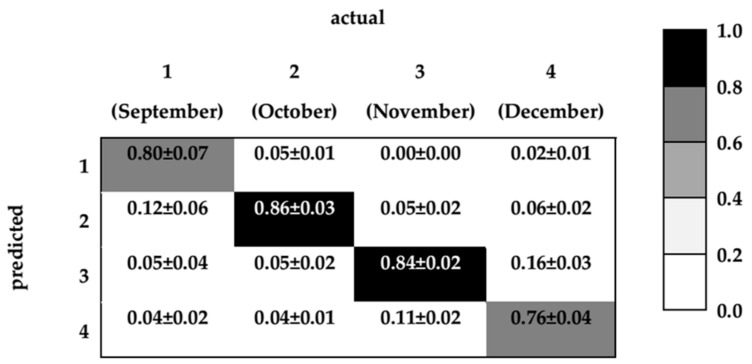
Confusion matrix obtained from the evaluation of ANN models for unseen data.

The input layer of final ANNs consisted of the heights of 15 selected peaks by using the SA. The output layer specified four classes of ripening stages. Figure 6 shows the performance of the constructed ANNs using the external validation data. The external validation data for each RS iteration consisted of 20 samples with three replicates each. External validation data was stratified per class.

Despite the complexity of the dataset, the overall accuracy of the model was obtained as 82 ± 1%. It is 20% higher than the average accuracy of models which consider all peaks as inputs. It means that the feature selection not only reduced the complexity of the models but also increased the accuracy. According to the confusion matrix, the mean classification accuracy of ANN models for the first, second, third, and fourth classes was 80 ± 7%, 86 ± 3%, 84 ± 2%, and 76 ± 4%, respectively. The confusion matrix (see Figure 6) shows the model could classify ripening stages of bitter orange EOs based on their extracted features of chromatograms obtained from HS-GC-MS analyses. As shown in the confusion matrix, the second and third stages were easier to classify. September samples were partially classified as October samples (12% of cases), and December samples were partially classified as November samples. We see that the errors appear just in consecutive months at the beginning and at the end of the ripening process. This may indicate a sigmoidal time evolution of the ripening process so that the most important changes occur in the central months, while the evolution is slower at the beginning and at the end of the ripening process. In fact, it is known that the evolution of volatile production can be strongly non-linear during the ripening process. For example, the intensity of β-pinene slightly decreases at first and then increases; while myrcene increases in the initial stages of ripening, but later it decreases and its amount in the initial and final stages of ripening is similar [8].

**Figure 7:**
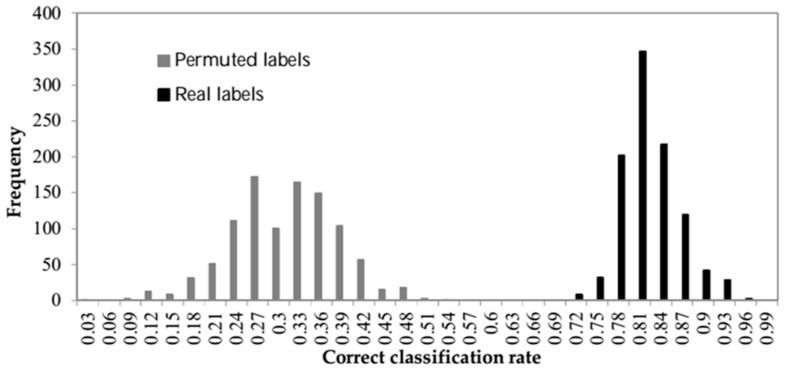
The histogram of correct classification rate of data with both real and permuted labels (null hypothesis distribution).

To confirm the statistical significance of the obtained results, a permutation test was used. The histogram of two sets of classification (with real and permuted labels) is shown in Figure 7. The Mann–Whitney U test [50] was used as a nonparametric test for equality of population medians of the two samples. The result rejects the null hypothesis at the 1% significance level. So, they are meaningfully different.

Thus, the ripening stages of bitter orange EOs were successfully classified based on chromatogram features obtained from HS-GC-MS analyses. This could help differentiating EOs from different ripening stages for their distinct applications. On the basis of sensitivity analysis, the peak heights of 15 compounds were evaluated as effective for the aforementioned purpose. These compounds are among those which were previously reported to have an important role in characterizing bitter orange EO [18]. However, limonene and myrcene, reported as the major compounds of bitter orange EO [7,11,12,52], were not among the effective peaks for the classification. In fact, Ahmed et al. [52] also reported that both compounds have a higher odor threshold when compared to α-pinene for example. From this point of view, it seems that the peaks detected by our analysis follow the perception impact of the different compounds. That is, the procedure automatically selects compounds that are relevant from a perceptual point of view.

Overall, the developed approach can be an effective step in creating an appropriate alternative to the subjective methods of quality assessment of EOs. This highly repeatable and reproducible method can overcome the mentioned disadvantages of sensory evaluation, such as susceptibility to large sources of variation and the time-consuming nature of it. Unlike sensory evaluation, a trained panel is not required. The combination of headspace chromatography and machine learning for classification increases both the speed and efficiency of the proposed quality assessment. Moreover, the need for an expert to design and manage the sensory evaluation and interpret the obtained data was eliminated. As for the limitations of the current approach, we should mention that the current prediction models could be location specific, taking into consideration the conditions of the used cultivars. The present study did not take into account the ability of this model to function for other bitter orange samples over diverse geographical origins. This would require sampling bitter oranges in different parts of the world and remains out of the scope of this initial study.

Classifying bitter orange EOs using the different ripening degrees based on consumer preference or other applications of constituents can be explored in the light of the obtained results.

## 4. Conclusions

The obtained results show that the ripening degree can be predicted from key volatile compounds in the bitter orange EO headspace using gas chromatography mass spectrometry, avoiding the need for sensorial analysis by trained human panels. The proposed methodology combines chromatographic fingerprinting followed by data analysis using machine learning techniques, in particular ANN. A non-linear classifier (e.g., an MLP) was used since the PCA of the data matrix did not show simple separation of the different classes using three PCs that accounted for 90% of the total variance. Additionally, sensitivity analysis was used to determine the chemotypes responsible for the observed differentiation. The analysis shows that the intensity of fifteen compounds is enough to classify the ripening degree in four classes with a moderate accuracy of 82 ± 1%. The statistical significance of this result was confirmed using a permutation test. The main compounds to predict the ripening degree of bitter orange are α-pinene, β-pinene, ocimene, Cyclopropane,1,2-dibutyl-, linalyl butyrate, myrcenol, linalool α-terpineol, 3-carne, and nerol. These results open a new approach to quality control for bitter orange EOs.

## Figures and Tables

**Table 1:** Chromatographic retention time (RT), possible identity, molecular weight (MW), and the aromatic note of the compounds identified in the headspace of bitter orange essential oil (EO).

No.	RT	Compounds *	Aromatic Note of EO	MW
1	1.99	ND	-	-
2	5.17	ND	-	-
3	6.95	α-pinene	Floral	136.24
4	8.27	β-pinene	Green	136.24
5	8.41	ND	-	-
6	8.83	β-myrcene	Green	136.24
7	9.43	ND	-	-
8	9.92	ND	-	-
9	10.45	limonene	Citrus	136.24
10	11.30	ocimene	Citrus	136.24
11	11.82	Cyclopropane,1,2-dibutyl-	-	154.30
12	12.48	cis-linalool oxide	Floral	170.25
13	13.28	myrcenol	-	154.25
14	13.80	linalool	Floral	154.25
15	15.64	ND	-	-
16	16.83	ND	-	-
17	18.16	linalyl butyrate	Floral	224.34
18	19.01	α-terpineol	Green	154.25
19	21.66	3-carne	Sweet; citrus	136.24
20	23.40	nerol	Floral	154.25
21	25.70	ND	-	-
22	26.07	ND	-	-

* identified in scan mode, ND = not determined.

**Table 2:** The ranking of peaks based on their sensitivity on predicting each class.

Output	Peaks Ranking																					
**First Class**	11	1	3	15	18	19	20	10	4	5	9	17	21	14	12	13	22	6	8	2	16	7
**Second Class**	11	19	18	10	3	20	4	22	15	13	17	1	9	21	12	5	8	14	2	6	16	7
**Third Class**	19	1	11	10	18	20	15	17	14	5	4	22	3	21	13	12	9	8	6	2	16	7
**Fourth Class**	1	19	11	18	10	20	15	22	21	17	5	3	14	4	13	9	6	12	16	2	8	7

**Table 3:** The inputs which were added in each step for feature selection.

**Sequence**	1	2	3	4	5	6	7	8	9	10	11	12	13
**No of Peaks (No. of ANN Inputs)**	3	5	7	8	9	11	13	15	16	17	19	21	22
**Peaks No. Added in Each Step**	1,11,19	3,18	10,15	20	4	17,22	14,21	5,13	9	12	6,8	2,16	7
